# Novel Coronavirus and Astrovirus in Delaware Bay Shorebirds

**DOI:** 10.1371/journal.pone.0093395

**Published:** 2014-04-03

**Authors:** Kirsi S. Honkavuori, Thomas Briese, Scott Krauss, Maria D. Sanchez, Komal Jain, Stephen K. Hutchison, Robert G. Webster, W. Ian Lipkin

**Affiliations:** 1 Center for Infection and Immunity, Mailman School of Public Health, Columbia University, New York, New York, United States of America; 2 Department of Infectious Diseases, St. Jude Children’s Research Hospital, Memphis, Tennessee, United States of America; 3 454 Life Sciences, Branford, Connecticut, United States of America; NIH, United States of America

## Abstract

**Background:**

Wild birds are an important but to some extent under-studied reservoir for emerging pathogens. We used unbiased sequencing methods for virus discovery in shorebird samples from the Delaware Bay, USA; an important feeding ground for thousands of migratory birds.

**Findings:**

Analysis of shorebird fecal samples indicated the presence of a novel astrovirus and coronavirus. A sanderling sample yielded sequences with distant homology to avian nephritis virus 1, an astrovirus associated with acute nephritis in poultry. A ruddy turnstone sample yielded sequences with homology to deltacoronaviruses.

**Conclusions:**

Our findings highlight shorebirds as a virus reservoir and the need to closely monitor wild bird populations for the emergence of novel virus variants.

## Introduction

Wild birds have been recognized as important reservoir hosts harboring and amplifying emerging zoonotic viruses such as avian influenza A viruses [1] and West Nile virus [2]. In the northeastern USA, the Delaware Bay area is crucial for the annual migration of shorebird and gull species that feed on the horseshoe crab eggs found in abundance thanks to the coinciding spawning season (for review see [3]). Avian influenza virus isolation rates from shorebirds and gulls during spring migration are significantly higher in this area than in other surveillance sites [3], [4], [5]. This observation prompted us to assess the ability to detect other, novel viruses in shorebirds during the spring migration.

## Materials and Methods

### Sample Collection

Shorebird fecal samples of apparently healthy birds were collected in May 2004 from Reed’s beach, Delaware Bay, New Jersey, USA, held at 4°C for shipment (≤96 h) and upon arrival at the laboratory stored at −80°C until further processing. Permission to enter restricted access beaches and obtain fecal droppings was granted annually by the State of New Jersey, Department of Environmental Protection, Division of Fish and Wildlife, Endangered and Nongame Species Program. The surveillance program was also approved by the St. Jude Children’s Research Hospital IACUC protocol #546.

### Pyrosequencing

Nucleic acid was extracted by using QIAamp Viral RNA Mini Kit (Qiagen, Hilden, Germany), treated with DNaseI (Ambion, Austin, TX, USA) and reverse transcribed using Superscript II kit (Invitrogen, Carlsbad, CA, USA) with random octamer primers linked to an arbitrary specific anchor sequence [6]. The cDNA was RNase H-treated prior to random amplification by the polymerase chain reaction (PCR). The resulting products were purified using MinElute (Qiagen, Hilden, Germany), pooled and ligated to linkers for sequencing on a GSL FLX Sequencer (454 Life Sciences, Branford, CT, USA). After primer and adaptor trimming, length filtering, masking of low complexity regions and subtraction of ribosomal and host sequences, sequence reads were assembled using Newbler Assembler (v 2.3, 454 Life Sciences) and analyzed at nucleotide (nt) and amino acid (aa) level by using homology search programs Blastn [7] and Fastx [8] against NCBI Refseq and GenBank databases (http://www.ncbi.nlm.nih.gov).

### Specific PCR and Assessment of Bird Species

Sequence-specific PCR was conducted with HotStar polymerase (Qiagen) and random hexamer-primed cDNA (Superscript II, Invitrogen). Amplified products were subjected to direct dideoxy sequencing (Genewiz, South Plainfield, NJ, USA). The bird species from which samples were obtained was identified by the nested PCR method of Cheung et al. that targets the mitochondrial cytochrome oxidase I (COI) gene [9].

### Phylogenetic Analysis

Assessment of phylogenetic relationships was performed using programs of the MEGA 5 software package (http://www.megasoftware.net). Multiple sequence alignments were generated with ClustalW, and phylogenetic trees constructed based on the neighbor joining method using a Poisson correction model to calculate distance; bootstrap values were calculated based on 1000 pseudoreplicates.

## Results and Discussion

We applied an unbiased pyrosequencing approach for analyzing total RNA extracts of fecal samples collected at Reed’s beach, Delaware Bay, New Jersey, USA, from apparently healthy shorebirds to assess potential microbial burden. Samples from eight individual birds, that included semipalmated sandpipers, sanderlings and ruddy turnstones, were extracted for total RNA, reverse transcribed and amplified by random PCR before the purified PCR products were pooled and processed for sequencing on the 454 pyrosequencing platform. Sequencing yielded 182,358 reads with a mean length of 320 nt (NIH Short Read Archive accession number SRP035900). After filtering for ribosomal and host sequences, the remaining 4171 sequences were processed to assemble possible contiguous sequences and then analyzed at nt and deduced aa level. Three sequences of 357, 468 and 474 nt length showed homology to members of the coronavirus (CoV) family and two sequences of 436 and 445 nt showed homology to avian astroviruses (AstV). In addition we detected reads with distant homology to tetnoviruses (2 reads) and marnavirus (1 read); viruses of insects and algae, respectively, that were in view of the limited sample material not further followed. No other sequences with significant homology to viral records in GenBank (release 175) were detected using an e-value cut-off of 0.001 for Blastn and Fastx.

The presence of the detected astro- and coronaviral sequences in individual samples that made up the pool of 8 used for pyrosequencing was confirmed by PCR assays using primers specific for the identified sequences (**Table S1**). Two of the eight samples pooled for pyrosequencing were confirmed to contain either CoV or AstV sequences. The CoV-positive fecal sample originated from a ruddy turnstone (*Arenaria interpres*) and the AstV-positive sample from a sanderling (*Calidris alba*), as determined by mitochondrial cytochrome oxidase I (COI) sequence analysis.

The sanderling derived AstV-related sequences assembled to an approximately 600 nt long continuous sequence that mapped to the middle region of the non-structural polyprotein 1a open reading frame (ORF). Analysis of a PCR product obtained with primers matching that sequence (GenBank accession number JX548301) indicated approximately 41% identity at aa level to avian nephritis virus 1 (ANV-1). Closer inspection of the recovered AstV sequence indicated similarity to trypsin-like peptidase family pfam13365 and to peptidase S7 superfamily pfam 00949 between aa residues 121–164 with conservation of the potential catalytic triad in the serine protease domain, including Histidine-24, Aspartic acid-61 and Serine-125 [10].

**Figure 1:**
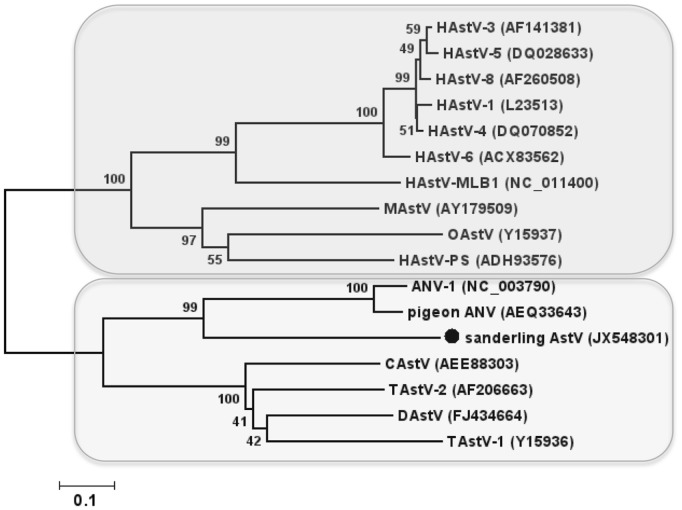
Phylogenetic analysis of sanderling astrovirus partial ORF1a sequence (198 aa, marked with a circle) and selected AstV sequences (indicated by their Genbank accession numbers in brackets). Bootstrap values based on 1000 pseudoreplicates are indicated at the respective nodes; scale bar indicates substitutions per site. The division of the family into *Mamastrovirus* genus and *Avastrovirus* genus is indicated by shaded boxes. HAstV, human AstV; OAstV, ovine AstV; MAstV, mink AstV; CAstV, chicken AstV; TAstV, turkey AstV; DAstV, duck AstV.

AstVs are small non-enveloped viruses with positive-sense, single-stranded RNA genomes. The family *Astroviridae* is divided into mammalian and avian AstVs (**Figure 1**) ([11], [12], http://www.iah-virus.org/astroviridae/avastrovirus). In addition to yet unassigned chicken AstVs, a novel wood pigeon AstV and feral pigeon AstVs were recently identified in Norway that appear to be highly divergent from ANV-1, their closest relative [13]. To gain further insight into the evolutionary relationship to other AstVs, a phylogenetic analysis of sanderling AstV was conducted based on a common 198 aa ORF1a sequence aligned to representative members of the family. The analysis indicated that sanderling AstV formed together with ANV-like AstV a branch distinct from other avian AstVs, namely those of duck, chicken and turkey origin (**Figure 1**). Because sequence information of ORF1a is only available for ANV-1 and pigeon ANV but not for the novel pigeon AstVs reported from Norway, we cannot determine whether sanderling AstV groups with the subgroup of pigeon AstVs suggested by Kofstad and Jonassen [13]. Attempts to obtain additional sequence flanking the 600 bp region failed. This may reflect compromised nucleic acid quality in the sample material in addition to possible primer failure due to high sequence divergence.

Avian AstVs are associated with enteric diseases in poultry [10], [14], [15], nephritis in chicken and turkeys [16], [17] and fatal hepatitis in ducklings [18]. ANV-like viruses as well as antibodies against them have been detected in both chicken and turkeys [17], [19], 20, suggesting that these viruses may successfully infect and replicate in different poultry species. The discovery of pigeon ANV that is related to chicken ANV supports the likelihood of cross-infection between pigeons and chicken [21], [22]. Additional evidence for interspecies transmission among avian AstVs was provided by the identification of guinea fowl AstV, that is related to turkey AstV type 2 [23], and the subsequent experimental infections conducted with both viruses in both avian species [24]. Sanderling AstV may potentially also be capable of infecting other avian species beyond its wild bird reservoir.

The sequences recovered from a ruddy turnstone mapped to ORF1ab, spike glycoprotein gene and the 3′ end of CoV genomes. Analysis of the 438 nt PCR-amplified spike glycoprotein gene sequence (GenBank accession number JX548305) suggested homology to the CoV S2 glycoprotein multidomain (pfam 01601). Further sequence analysis at aa level revealed 79% identity to magpie-robin CoV HKU18 and sparrow CoV HKU17, both identified in Hong Kong [25]. Analysis of the amplified 450 nt ORF1ab fragment (GenBank accession number JX548303) indicated approximately 70% identity at aa level to common-moorhen CoV HKU21, also detected in Hong Kong [25]. Analysis of the amplified 312 nt fragment (GenBank accession number JX548302) mapping partly to the 3′ untranslated region (UTR) also revealed relatedness to the CoVs recently identified by Woo and others [25] by showing 90% identity with white-eye CoV HKU16 3′UTR nt 200–312. A portion of the 312 nt fragment maps to the region potentially coding for nonstructural accessory proteins Ns7c or Ns7d.

**Figure 2:**
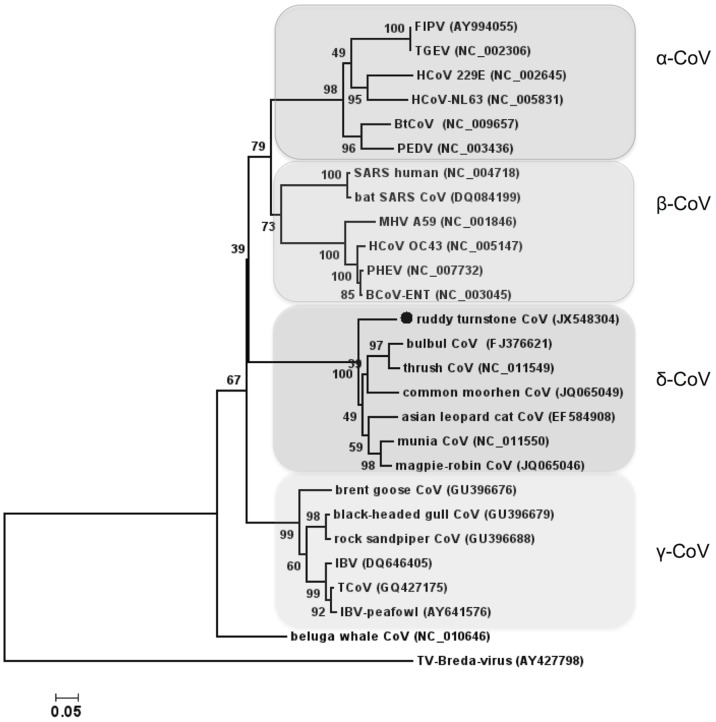
Phylogenetic analysis of ruddy turnstone CoV partial polymerase sequence (168 aa, marked with a circle) and selected CoV sequences (indicated by their Genbank accession numbers in brackets). Bootstrap values based on 1000 pseudoreplicates are indicated at the respective nodes; scale bar indicates substitutions per site. The division of the CoVs into alpha, beta, gamma and delta groups is indicated by shaded boxes. Breda virus, a torovirus from the subfamily *Torovirinae*, family *Coronaviridae* was used as an outlier. FIPV, feline infectious peritonitis virus; TGEV, transmissible gastroenteritis CoV; BtCoV, bat CoV; PEDV, porcine epidemic diarrhea virus; SARS, severe acute respiratory syndrome; MHV, mouse hepatitis virus; PHEV, porcine hemagglutinating encephalomyelitis virus; HCoV, human CoV; BCoV, bovine CoV; TCoV, turkey CoV; IBV, infectious bronchitis virus.

CoVs are enveloped RNA viruses with large, positive-sense single-stranded genomes. Members of the family *Coronaviridae* cause enteric, respiratory and central nervous system (CNS) diseases in a wide range of animal species [26], [27]. The subfamily *Coronavirinae* has been divided into four groups (**Figure 2**). Recent studies have revealed the presence of diverse, novel gamma and delta-CoVs in wild birds from Hong Kong [25], [28], Cambodia [28] and from the Bering Strait area [29]. Since full-length genomic sequences are not available for all of them, we assessed the relationship between those CoVs and ruddy turnstone CoV by amplifying a fragment of the polymerase (pol) gene with degenerate primers described by Muradrasoli et al. [29]. Phylogenetic analysis of the amplified 508 nt pol sequence (GenBank accession number JX548304) agreed with that of the partial spike, ORF1ab and 3′end (NS7c-like) sequences and showed a relation to the delta-CoV clade (**Figure 2**). The pol sequence of ruddy turnstone CoV shares approx. 86% aa identity with common-moorhen CoV HKU21 from Hong Kong [25], and is less related to the IBV-like viruses from the Bering Strait area that fall into the gamma-CoV cluster. As with Sanderling AstV, attempts to obtain additional sequences from ruddy turnstone CoV were unsuccessful.

Our findings indicate the importance of surveying wild bird populations for circulating viruses; the discovery of novel shorebird CoV and AstV sequences in a small sample set demonstrates the potential diversity of novel avian viruses yet to be identified. The phylogenetic relation of sanderling AstV to ANV-1 may indicate a potential for interspecies transmission between wild birds and poultry also suggested by others [21], [22], [23], [24]. The CoV sequences reported here along with recent discoveries by others [28], [29] imply a vast diversity among circulating avian CoVs and a high prevalence in wild birds [28]. Finally, the Delaware Bay area could serve as a useful surveillance site for such novel avian viruses, in addition to influenza viruses. Due to the high volume of birds feeding during migration periods, the area may provide a niche for the emergence and spread of novel virus variants.

## Supporting Information

Table S1 Oligonucleotides used in the study.
(DOCX)
