# Supplementary material for: Novel Coronavirus and Astrovirus in Delaware Bay Shorebirds
Source: PLoS One. 2014 Apr 3;9(4):e93395. doi: 10.1371/journal.pone.0093395 (PMC3974748; doi:10.1371/journal.pone.0093395)
Supplement: Table S1 — Oligonucleotides used in the study. (DOCX) [file pone.0093395.s001.docx]

**Table S1.** Oligonucleotides used in the study.

| Name | Target | Sequence |
| --- | --- | --- |
| CoVspikeforward | Coronavirus spike gene | 5’- GTTTGCAGAGCAACATAATTAAGTC -3’ |
| CoVspikereverse | Coronavirus spike gene | 5’- AGTGCCTGTTTCAGTATAGAACAAGC -3’ |
| CoVORF1abforward | Coronavirus ORF1ab | 5’- ATGCTCGTCATTTGCAAGTTTGG -3’ |
| CoVORF1abreverse | Coronavirus ORF1ab | 5’- ACATCCCATCTCACCAATAGACTG -3’ |
| CoVNS7cforward | Coronavirus ORF1ab | 5’- GTATACTCCATACCTTTCCCTCAATTC -3’ |
| CoVNS7creverse | Coronavirus ORF1ab | 5’- TGTCGTGGCCTCGGTTCAATTAATAAGG -3’ |
| CoVpolforward^§^ | Coronavirus polymerase | 5’- TGGGWTGGGAYTAYCCWAARTGYGA -3’ |
| CoVpolreverse^§^ | Coronavirus polymerase | 5’- GCATWGTRTGYTGNGARCARAATTC -3’ |
| AstV-ORF1a592forward | Astrovirus ORF1a | 5’- TGTTCTACATTGAGGTAACTTTGGA -3’ |
| AstV-ORF1a728reverse | Astrovirus ORF1a | 5’- GTGTATTGCAACAACTTTTCCA -3’ |
| AstV-ORF1a694forward | Astrovirus ORF1a | 5’- TGTTCCACTTTGGCTAGAAGCG -3’ |
| AstV-ORF1a800reverse | Astrovirus ORF1a | 5’- AGACTTGCAACGAGGACAGCGTGTG -3’ |
| ExternalF1† | Mitochondrial COI gene | 5’- TGTAAAAAGGWCTACAGCCTAACGC -3’ |
| ExternalR1† | Mitochondrial COI gene | 5’- GTRGCNGAYGTRAARTATGCTCG -3’ |
| InternalF1† | Mitochondrial COI gene | 5’- AACAAACCACAAAGATATCGG -3’ |
| InternalR1† | Mitochondrial COI gene | 5’- TGGGARATAATTCCRAAGCCTGG -3’ |

^§^Oligonucleotides from Muradrasoli et al.: [**Prevalence and phylogeny of coronaviruses in wild birds from the Bering Strait area (Beringia).**](http://www.ncbi.nlm.nih.gov/pubmed/21060827) *PLoS One* 2010, **5:**e13640.

†Oligonucletides from Cheung et al.: **Identifying the species-origin of feacal droppings used for avian influenza virus surveillance in wild-birds**. *J Clin Virol* 2009, **46**:90-93.
